# The Role of qSOFA, Derived Neutrophil-to-Lymphocyte Ratio, MEWS, and PIRO Scores in Predicting the Severity of Odontogenic Infections in Young and Adult Patients

**DOI:** 10.3390/biomedicines13030532

**Published:** 2025-02-20

**Authors:** Serban Talpos Niculescu, Robert Avramut, Tareq Hajaj, Raluca Maracineanu, Antonis Perdiou, Roxana Talpos Niculescu, Marius Pricop, Horatiu Urechescu, Florin Urtila, Roxana Radu, Nicoleta Nikolajevic Stoican, Malina Popa

**Affiliations:** 1 Discipline of Oral and Maxillo-Facial Surgery, Faculty of Dental Medicine, “Victor Babes” University of Medicine and Pharmacy Timisoara, Eftimie Murgu Square 2, 300041 Timisoara, Romania; talpos.serban@umft.ro (S.T.N.); pricop.marius@umft.ro (M.P.); urechescu.horatiu@umft.ro (H.U.); urtila.florin@umft.ro (F.U.); 2 Doctoral School, “Victor Babes” University of Medicine and Pharmacy Timisoara, Eftimie Murgu Square 2, 300041 Timisoara, Romania; robert.avramut@umft.ro (R.A.); raluca.zibileanu@umft.ro (R.M.); antonis.perdoiu@umft.ro (A.P.); stoican.nicoleta@umft.ro (N.N.S.); 3 Discipline of Prostheses Technology and Dental Materials, Faculty of Dental Medicine, “Victor Babes” University of Medicine and Pharmacy Timisoara, Eftimie Murgu Square 2, 300041 Timisoara, Romania; tareq.hajaj@umft.ro; 4 Discipline of Odontotherapy-Endodontics, Faculty of Dental Medicine, “Victor Babes” University of Medicine and Pharmacy Timisoara, Eftimie Murgu Square 2, 300041 Timisoara, Romania; clinci.roxana@umft.ro; 5 Stomatology Clinic, 310025 Arad, Romania; 6 Pediatric Dentistry Research Center (Pedo-Research), Department of Pediatric Dentistry, Faculty of Dental Medicine, “Victor Babes” University of Medicine and Pharmacy Timisoara, Eftimie Murgu Square 2, 300041 Timisoara, Romania; popa.malina@umft.ro

**Keywords:** dental infections, sequential organ failure assessment score, neutrophil–lymphocyte ratio, early warning systems, prognostic factors, inflammatory mediators, adult, adolescent

## Abstract

**Background and Objectives:** Odontogenic infections (OIs) can lead to severe complications if not promptly diagnosed and treated. The Quick Sequential Organ Failure Assessment (qSOFA), derived Neutrophil-to-Lymphocyte Ratio (dNLR); Modified Early Warning Score (MEWS); and Predisposition, Infection, Response, and Organ Dysfunction (PIRO) scores are clinical tools used to predict the severity and outcomes in various infections. This study aims to evaluate the efficacy of these scores in predicting the severity of OIs in adult patients. **Methods:** A retrospective cohort study was conducted on 120 patients hospitalized for OIs, divided into two groups based on infection severity, using the Symptom Severity (SS) scale. The qSOFA, dNLR, MEWS, and PIRO scores were calculated upon admission. Statistical analyses were performed to assess the predictive value of these scores for severe OIs. **Results:** Patients with severe OIs (Group B) had significantly higher qSOFA, dNLR, MEWS, and PIRO scores compared to those with lower severity (Group A). The median qSOFA score was 2.00 in Group B versus 0.85 in Group A. No significant difference was observed between young patients and adults in terms of severity. ROC curve analysis showed that the PIRO score had the highest predictive value for severe OI (AUC = 0.912), followed by MEWS (AUC = 0.878), qSOFA (AUC = 0.845), and dNLR (AUC = 0.812). Multivariate logistic regression indicated that the PIRO score was an independent predictor of severe OI (OR = 8.45, 95% CI: 4.12–12.78). **Conclusions:** The qSOFA, dNLR, MEWS, and PIRO scores are valuable tools for predicting the severity of OIs. Among them, the PIRO score demonstrated the highest predictive accuracy and may be incorporated into clinical practice for early identification of high-risk patients.

## 1. Introduction

Odontogenic infections (OIs) represent a significant portion of dental and maxillofacial emergencies, often resulting from delayed presentations or misdiagnosed complications. Despite advances in dental care and antibiotic therapy, OIs can progress rapidly, leading to severe systemic involvement and life-threatening conditions if not promptly recognized and managed [1,2].

Early identification of patients at risk for severe outcomes is crucial for optimizing treatment strategies and improving prognosis, whether in adults or younger patients, such as children and adolescents. Traditionally, clinicians have relied on clinical assessments and basic laboratory parameters to gauge the severity of OIs. However, these methods may lack sensitivity and specificity, and their effectiveness can vary across different age groups, underscoring the need for reliable predictive tools applicable to all patients [3,4].

Several scoring systems have been developed to assess the severity of infections and predict patient outcomes. The Quick Sequential Organ Failure Assessment (qSOFA) score is a simplified version of the SOFA score, used to identify patients with suspected infection who are at greater risk for poor outcomes outside the intensive care unit [5]. The derived Neutrophil-to-Lymphocyte Ratio (dNLR) is an easily obtainable inflammatory marker calculated from routine blood counts, serving as an indicator of systemic inflammation [6].

Recent studies have highlighted the utility of blood count-derived markers, such as the NLR, in diagnosing and assessing the severity of various infections. For instance, it was demonstrated that NLR, alongside other inflammatory markers, effectively differentiates periprosthetic joint infections from aseptic failures in total hip arthroplasties, showcasing its high sensitivity and independent diagnostic value [7]. These findings underscore the potential of NLR as a reliable indicator of systemic inflammation and infection severity. Incorporating NLR into the evaluation of OI could enhance the accuracy of severity predictions, allowing for more timely and targeted interventions in both adult and adolescent populations.

The Modified Early Warning Score (MEWS) is a bedside tool used to detect early signs of patient deterioration by assessing vital parameters [8,9]. The Predisposition, Infection, Response, and Organ Dysfunction (PIRO) score is a comprehensive staging system that evaluates multiple dimensions of a patient’s condition to predict outcomes in sepsis [10]. While these scores have been validated in various clinical settings, their applicability in predicting the severity of OIs remains underexplored.

In this study, we aim to evaluate the efficacy of the qSOFA, dNLR, MEWS, and PIRO scores in predicting the severity of OIs in adolescent and adult patients. By determining their predictive value, we hope to enhance clinical decision-making and improve patient outcomes in OI management.

## 2. Materials and Methods

### 2.1. Study Design and Ethics

This retrospective cohort study was conducted at the Maxillofacial Surgery Department of the City Emergency Hospital Timisoara (SCMUT), affiliated with the Victor Babes University of Medicine and Pharmacy, Timisoara, Romania. The study period spanned from January 2018 to June 2023. Ethical approval was obtained from the SCMUT Ethics Committee (approval I-27098 from 14 October 2022), and the study adhered to the Declaration of Helsinki principles. Patients’ data were collected from electronic medical records after obtaining informed consent. All patient information was anonymized and de-identified prior to analysis to maintain confidentiality.

The study protocol was approved by the Institutional Review Board, and written informed consent was obtained from all participants or their legal guardians. The study adhered to the ethical standards of the Declaration of Helsinki, the EU Good Clinical Practice Directives (2005/28/EC), and the International Council for Harmonization of Technical Requirements for Pharmaceuticals for Human Use (ICH) guidelines.

During the preparation of this work, the authors used ChatGPT (OpenAI, San Francisco, CA, USA) in order to exclusively improve the manuscript’s language and readability. After using this tool, the authors reviewed and edited the content as needed, and they take full responsibility for the content of the publication. All the scientific content, interpretations, and conclusions are the original work of the authors.

### 2.2. Patient Selection and Study Groups

To justify the inclusion of patients aged 14 years and older, we adhered to the World Health Organization (WHO)’s age classifications, which categorize individuals aged 10–19 years as adolescents. By setting the lower age limit at 14, we ensured that the study population encompassed mid-adolescents and adults, thereby aligning with developmental and physiological considerations relevant to OIs [11]. This age threshold allows for a more homogeneous analysis of infection severity and response to treatment across a broader age spectrum. Patients between 10 and 13 years old were not included due to the fact that the hospital does not admit younger patients. OIs were identified using the International Classification of Diseases (ICD-10) codes [12]. Exclusion criteria included incomplete medical records, pregnancy, immunodeficiency disorders, malignancies, and infections of non-OI. A total of 120 patients were included in the final analysis. Patients were divided into two groups based on the Symptom Severity (SS) score, ranging from 0 to 19: Group A (low severity, SS score ≤ 8) and Group B (high severity, SS score ≥ 9)).

### 2.3. Data Collection and Variables

Demographic data, clinical presentation, laboratory results, and outcomes were extracted from medical records. Clinical variables included vital signs; signs of systemic infection; and symptoms such as trismus, dysphagia, and fascial space involvement. Laboratory parameters collected upon admission included complete blood count (CBC), neutrophil and lymphocyte counts, and other routine blood tests. The qSOFA, dNLR, MEWS, and PIRO scores were calculated for each patient based on admission data.

### 2.4. Outcomes and Outcome Measures

The Symptom Severity score (SS) for OI is outlined with a maximum achievable score of 20 [13]. The scoring for Systemic Inflammatory Response Syndrome (SIRS) criteria [14] is as follows: 1 point for a temperature above 38.3 °C, 1 point for a heart rate exceeding 90 bpm, 1 point for a respiratory rate of 20/min or more, and 1 point for a white blood cell (WBC) count under 4 or over 12 × 10^9^, reaching a total maximum of 4 points. Trismus severity affects scoring, with moderate trismus (mouth opening less than 2 cm) receiving 3 points and severe trismus (mouth opening less than 1 cm) receiving 4 points, also up to a total of 4 points. Dysphagia is rated as mild, allowing the swallowing of most foods for 2 points, moderate for an inability to swallow fluids at 4 points, and severe for drooling saliva at 5 points, adding up to a total maximum of 5 points. The scoring for the presence of a collection in one facial space varies by severity: low severity (canine, vestibular) earns 1 point, moderate severity (buccal) earns 2 points, and high severity (all other spaces) earns 4 points, with a possible total of 5 points. A collection spanning two or more facial spaces immediately receives 5 points. Signs of dehydration, which are marked by low blood pressure (BP), increased urea, or reduced skin turgor, contribute 1 point to a possible 2. Additionally, the presence of comorbidities such as diabetes mellitus, immunocompromised status, or chronic alcohol misuse adds 1 point.

qSOFA score: The qSOFA score assesses the risk of poor outcomes in patients with suspected infection. It includes three criteria: systolic blood pressure ≤ 100 mm Hg, respiratory rate ≥ 22 breaths per minute, and altered mental status (Glasgow Coma Scale < 15). Each positive criterion scores one point, with a total score ranging from 0 to 3 [15].

Derived Neutrophil-to-Lymphocyte Ratio (dNLR): The dNLR is calculated by dividing the absolute neutrophil count by the difference between the white blood cell count and the neutrophil count: dNLR = Neutrophils/(WBC − Neutrophils). It reflects systemic inflammation, with higher values indicating greater inflammatory response [16].

Modified Early Warning Score (MEWS): The MEWS assesses the risk of clinical deterioration by evaluating vital signs: systolic blood pressure, heart rate, respiratory rate, temperature, and level of consciousness. Each parameter is scored from 0 to 3, resulting in a total score ranging from 0 to 14. Higher scores indicate increased risk [17].

PIRO score: The PIRO scoring system evaluates four domains: Predisposition (comorbidities), Infection (type and site), Response (physiological and laboratory markers), and Organ Dysfunction. Each domain is scored based on specific criteria, with the total PIRO score ranging from 0 to 20. Higher scores indicate greater severity and risk of adverse outcomes [18].

### 2.5. Statistical Analysis

Aiming for a statistical power of 80%, with a significance level of α = 0.05, it was determined that a minimum of 90 patients would be required. At the end of the data collection, a total of 120 patients were identified as eligible to be included in the current study. This sample size, evenly divided into two groups of 60 patients each (low severity and high severity), allows for a reliable comparison of the four scoring systems using logistic regression analysis. G*Power (Universität Kiel, Kiel, Germany) was used for statistical power and sample size calculation. Data were analyzed using SPSS version 25.0 (IBM Corp., Armonk, NY, USA). Continuous variables were expressed as mean ± standard deviation or median with interquartile range, depending on normality. Categorical variables were presented as frequencies and percentages. Comparisons between groups were made using Student’s *t*-test or Mann–Whitney U test for continuous variables, and Chi-square or Fisher’s exact test for categorical variables. Receiver Operating Characteristic (ROC) curve analysis was performed to assess the predictive value of each score. Additionally, for the statistical analysis, we performed normality assessments using the Shapiro–Wilk test to determine the distribution of continuous variables. Multivariate logistic regression was used to identify independent predictors of severe OI. A *p*-value < 0.05 was considered statistically significant.

## 3. Results

### 3.1. Patient Characteristics

The mean age of patients in Group A was significantly lower, at 45.82, years compared to 52.36 years in Group B (*p* = 0.024). A higher percentage of patients from Group A originated from urban areas (53.33% vs. 36.67%, *p* = 0.041), whereas Group B had a larger proportion of smokers (40.00% vs. 20.00%, *p* = 0.029) and individuals with diabetes mellitus (43.33% vs. 16.67%, *p* < 0.001). There were no significant differences between the groups regarding gender distribution, obesity, chronic kidney disease, or hypertension, as presented in Table 1.

### 3.2. Infection Characteristics

In the evaluation of infection characteristics among patients with OI, significant disparities were found between Group A and Group B concerning the type of infection, outcomes related to sepsis, ICU admission, and the duration of hospitalization. Group A primarily presented with abscesses (70.00%) compared to just 30.00% in Group B, while Group B exhibited a higher incidence of both abscesses and cellulitis simultaneously (50.00% vs. 16.67%; *p* < 0.001). Although the differences in infection sites were not statistically significant, notable differences were seen in patient outcomes. Sepsis occurred more frequently in Group B (25.00% vs. 8.33%, *p* = 0.012), as did ICU admissions (10.00% vs. 1.67%, *p* = 0.049). Additionally, the duration of hospitalization was significantly longer in Group B, averaging 11.84 days compared to 4.56 days in Group A (*p* < 0.001). Mortality was higher in Group B, although it did not reach statistical significance (5.00% vs. 0.00%, *p* = 0.079), as presented in Table 2.

### 3.3. Symptom Severity Scores

The SIRS score significantly varied, with Group A having more patients with lower scores (0 and 1), and Group B displaying a higher proportion of patients with severe systemic inflammation (scores of 3 and 4; *p* < 0.001). Similarly, trismus severity was notably different, with 56.67% of Group A presenting as normal compared to only 20.00% in Group B, while 50.00% of Group B experienced severe trismus versus 10.00% in Group A (*p* < 0.001). Dysphagia scores also differed significantly, with Group B having twice the proportion of patients with moderate dysphagia compared to Group A (50.00% vs. 30.00%; *p* = 0.018). The fascial space involvement showed that 66.67% of patients in Group A were at low risk, compared to only 23.33% in Group B, who had a higher percentage at high risk (30.00% vs. 3.33%; *p* < 0.001). Moreover, dehydration or comorbidities affected 33.33% of Group B in combination, compared to just 6.67% in Group A (*p* = 0.002), as seen in Table 3.

### 3.4. Laboratory and Biomarker Scores

The mean white blood cell (WBC) count was significantly higher in Group B (13.72 × 10^9^/L) compared to Group A (9.85 × 10^9^/L, *p* < 0.001). Similarly, neutrophil counts were elevated in Group B (10.24 × 10^9^/L) versus Group A (6.58 × 10^9^/L, *p* < 0.001), while lymphocyte counts were lower in Group B (1.74 × 10^9^/L) compared to Group A (2.12 × 10^9^/L, *p* = 0.002). The median derived Neutrophil-to-Lymphocyte Ratio (dNLR) and the Quick Sequential Organ Failure Assessment (qSOFA) score were substantially higher in Group B (6.20 and 2.00, respectively) compared to Group A (2.85 and 0.85, respectively; *p* < 0.001 for both). The Modified Early Warning Score (MEWS) and PIRO (Predisposition, Infection, Response, and Organ Dysfunction) score also showed significant disparities, with Group B having much higher mean scores (7.84 and 14.56) than Group A (3.22 and 6.18, *p* < 0.001), as presented in Table 4.

### 3.5. Predictive Value of Scoring Systems

The Quick Sequential Organ Failure Assessment (qSOFA) score demonstrated a strong association with severe infections, with an odds ratio (OR) of 5.68 (95% CI: 3.28–8.72, *p* < 0.001). Similarly, the derived Neutrophil-to-Lymphocyte Ratio (dNLR) was also a significant predictor, with an OR of 4.22 (95% CI: 2.86–6.54, *p* < 0.001). The Modified Early Warning Score (MEWS) showed an even higher predictive value, with an OR of 6.45 (95% CI: 3.78–9.12, *p* < 0.001). The highest predictive strength was observed with the PIRO (Predisposition, Infection, Response, and Organ Dysfunction) score, which had an OR of 8.45 (95% CI: 4.12–12.78, *p* < 0.001), as seen in Table 5.

**Figure 1:**
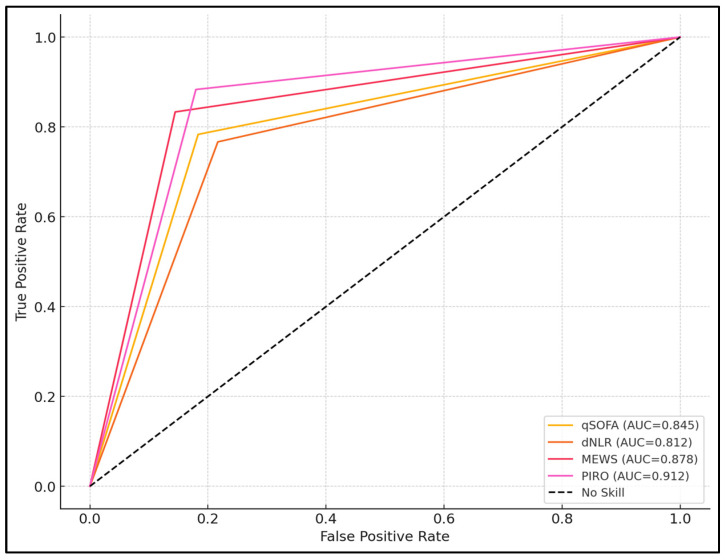
ROC curve analysis of scoring systems.

The Quick Sequential Organ Failure Assessment (qSOFA) score had an area under the curve (AUC) of 0.845, demonstrating high accuracy, with 78.33% sensitivity and 81.67% specificity, thus highlighting its effectiveness in predicting severe outcomes (*p* < 0.001). The derived Neutrophil-to-Lymphocyte Ratio (dNLR) also showed substantial predictive capability, with an AUC of 0.812, sensitivity of 76.67%, and specificity of 78.33% (*p* < 0.001). The Modified Early Warning Score (MEWS) exhibited a higher AUC of 0.878, alongside an impressive sensitivity of 83.33% and specificity of 85.61%, confirming its utility in clinical assessments (*p* < 0.001). The highest diagnostic accuracy was observed with the PIRO (Predisposition, Infection, Response, and Organ Dysfunction) score, which achieved an AUC of 0.912, with 88.33% sensitivity and 82.05% specificity, making it the most reliable scoring system among those tested for predicting severe OI (*p* < 0.001), as presented in Table 6 and Figure 1.

## 4. Discussion

### 4.1. Analysis of Findings

The study demonstrated that the qSOFA, dNLR, MEWS, and PIRO scores each played a crucial role in predicting the severity of OIs. These findings were significant, showing high statistical associations and differential capabilities across the scoring systems. The qSOFA and dNLR were particularly notable for their operational simplicity and potential to be implemented rapidly in clinical settings, characteristics that are crucial for early intervention in severe infection cases. However, while both showed strong predictive values, the PIRO score’s higher OR and AUC suggested it might be more effective in identifying patients at greatest risk of severe complications.

Further analysis revealed that the MEWS and PIRO scores not only provided high sensitivity and specificity but also highlighted the importance of a comprehensive scoring system that incorporates multiple aspects of a patient’s physiological state. The PIRO score, which includes components such as predisposition, infection characteristics, response to infection, and organ dysfunction, offered a more detailed predictive framework. This suggests that the complexity of the scoring system might enhance the predictive accuracy, which is vital for managing severe infections effectively.

The utility of these scoring systems extends beyond simple prediction; they potentially allow for stratification of patients into risk categories, enabling more tailored therapeutic interventions. This stratification could lead to better resource allocation in healthcare settings, ensuring that patients with severe OI receive the necessary care promptly, thereby potentially reducing hospital stays and improving overall outcomes.

In a similar manner, the study by Eun-Sung Kang et al. [19] and Ji-Kwan Kim et al. [20] explored the diagnostic value of specific biomarkers in predicting the severity of OIs. Kang et al. focused on the role of presepsin (PSEP), alongside traditional markers, such as C-reactive protein (CRP), white blood cell count (WBC), and procalcitonin (PCT), finding that PSEP, with a cutoff value of 671.5 pg/mL, significantly correlates with sepsis in OIs. They reported moderately positive correlations between CRP and PCT, CRP and PSEP, and PCT and PSEP levels, indicating their combined utility in early emergency assessments. Conversely, Kim et al. highlighted the efficacy of procalcitonin alone, with an area under the curve (AUC) of 0.927 and a calculated cutoff value of 0.87 ng/mL for distinguishing between sepsis and non-sepsis in maxillofacial infections, underscoring its superior predictive power compared to traditional tests, like WBC and CRP.

Moreover, Liu et al. [21] assessed the combination of systemic immune-inflammation index (SII) with qSOFA and found that this combination predicted 28-day mortality in sepsis patients with a high area under the receiver operating characteristic (AUROC) of 0.840. They established an optimal SII cutoff value of >1.7668 as a high-risk indicator for mortality, significantly enhancing the predictive power of qSOFA alone. Meanwhile, Bond et al. [22] examined the relationship between OI locations and sepsis risk, discovering that deep OI significantly increased the likelihood of a higher qSOFA score, with a relative risk of 5.4 times greater than superficial OI.

Tarle et al. [23] introduced the Aggregate Index of Systemic Inflammation (AISI) and demonstrated its superiority over traditional markers like C-reactive protein (CRP) in predicting the severity of odontogenic abscesses. AISI achieved a higher sensitivity (82.93%) and specificity (81.63%) with an area under the curve (AUC) of 0.90, compared to an AUC of 0.74 for CRP, indicating a more effective prognostic capability in assessing severe infections. Conversely, Kabanova et al. [24] assessed the incidence of Systemic Inflammatory Response Syndrome (SIRS) and procalcitonin levels in patients with maxillofacial OI, finding a significant increase in procalcitonin levels correlated with the severity of infection and SIRS development. They reported that procalcitonin levels were significantly higher in patients with more severe infections; for example, levels reached 0.41 pg/mL in cases of Ludwig’s angina compared to 0.034 pg/mL in less severe cases.

Similarly to our study, the studies by Junya Kusumoto et al. [25] and Nathalie Pham Dang et al. [26] examined the use of hematologic and inflammatory parameters to determine the severity and prognosis of OI, highlighting the importance of specific markers in clinical decision-making. Kusumoto et al. [25] focused on the utility of routine blood tests and specific indices, like the Laboratory Risk Indicator for Necrotizing Fasciitis (LRINEC) score and the combined C-reactive protein (CRP) + Neutrophil-to-Lymphocyte Ratio (NLR), in differentiating between severe infection groups, such as deep neck abscess and necrotizing soft tissue infection (NSTI). They found that a cutoff value of 27 for CRP + NLR was particularly effective for identifying more severe cases, with the systemic immune-inflammation index (SII) providing a classification accuracy of 89.3% for severe cases when combined with CRP + NLR thresholds. Similarly, Pham Dang et al. [26] utilized clinical criteria and routine blood markers to segregate patients into groups requiring simple versus complex management, identifying factors such as penicillin allergy, psychiatric disorders, and specific edemas as predictors of complex outcomes.

The integration of systemic inflammation markers and clinical scoring systems is crucial in predicting the severity of OIs, as demonstrated by our study on qSOFA, derived Neutrophil-to-Lymphocyte Ratio (NLR), MEWS, and PIRO scores. The scoping review by Cecoro et al. [27] underscores the significant bidirectional relationship between periodontitis and low-grade inflammation (LGI), which not only exacerbates oral infections but also links to systemic chronic diseases, highlighting the importance of comprehensive inflammatory assessment in odontogenic cases. Complementarily, Tarle et al.’s comparative study reveals that the Aggregate Index of Systemic Inflammation (AISI) outperforms traditional markers like CRP in predicting abscess severity, suggesting that incorporating such indices alongside our predictive scores could enhance clinical accuracy [23]. Additionally, Miller et al. [28] demonstrate that markers like myeloperoxidase (MPO) are sensitive indicators of vascular inflammation related to tooth infections, reinforcing the need for multifaceted inflammatory evaluations in managing severe OIs. Furthermore, Hoare et al. [29] illustrate the potential long-term implications of chronic oral inflammation on carcinogenesis, emphasizing the broader significance of accurately predicting and managing infection severity to mitigate systemic health risks. Collectively, these studies support the multifaceted approach of our research, advocating for the integration of advanced inflammatory markers and clinical scoring systems to improve prognostic accuracy and patient outcomes in OI.

From a clinical utility perspective, these scoring systems, particularly PIRO, can significantly enhance the clinical management of OI. By enabling early identification of patients at high risk of severe outcomes, these tools support timely and appropriate clinical interventions. This early stratification can aid in optimizing the use of medical resources, improving patient outcomes, and potentially reducing the economic burden on healthcare systems.

Assessing the cost-effectiveness of predictive scores is essential given the necessity of blood collection and laboratory analyses in our study. Among the evaluated scores—qSOFA, NLR, MEWS, and PIRO—the NLR stands out as the most economical option, as it relies on standard complete blood count parameters that are routinely available and inexpensive to obtain. This makes NLR a practical choice for widespread clinical use without imposing significant additional costs. Additionally, it is important to note that blood collection was performed for all admitted patients not because they had simple dental infections, but because admissions were reserved for those exhibiting signs and concerns for sepsis. This selective approach ensures that laboratory resources are directed towards patients with a higher risk of severe systemic complications, thereby optimizing both clinical outcomes and economic efficiency in managing OIs.

### 4.2. Study Limitations

Despite the robust findings, this study has several limitations that warrant mention. Firstly, its retrospective design may introduce biases related to data collection and selection, as the analysis was confined to available records and predefined clinical parameters, potentially overlooking subtle clinical nuances that could affect the outcomes. Secondly, the study was conducted in a single center, which may limit the generalizability of the results to other settings with different patient populations or healthcare practices. Thirdly, the exclusion criteria might have eliminated certain complex cases that could provide further insights into the predictive power of the scoring systems used. This selective sampling might limit the generalizability of the study findings to the broader population of patients with dental infections, as those included in the study are more likely to have severe presentations. These factors suggest that the findings, while promising, should be interpreted with caution and validated through prospective, multicentric studies to confirm their applicability across diverse clinical environments.

## 5. Conclusions

In conclusion, the findings from this study confirm the high predictive accuracy of the qSOFA, dNLR, MEWS, and PIRO scores for assessing the severity of OIs, without significant differences identified among age groups. Among these, the PIRO score was the most effective, suggesting that its comprehensive approach in incorporating various aspects of an infection makes it a superior tool for predicting severe outcomes.

## Figures and Tables

**Table 1:** Demographic and clinical characteristics of patients with OI.

Variables	Group A (*n* = 60)	Group B (*n* = 60)	*p*-Value
Age (years), mean ± SD	45.82 ± 16.54	52.36 ± 17.28	0.024
Age range	14–63	16–65	
Gender, *n* (%)			0.317
Male	34 (56.67%)	38 (63.33%)	
Female	26 (43.33%)	22 (36.67%)	
Place of origin, *n* (%)			0.041
Urban	32 (53.33%)	22 (36.67%)	
Rural	28 (46.67%)	38 (63.33%)	
Smoking status, *n* (%)			0.029
Yes	12 (20.00%)	24 (40.00%)	
No	48 (80.00%)	36 (60.00%)	
Comorbidities, *n* (%)			
Diabetes mellitus	10 (16.67%)	26 (43.33%)	<0.001
Obesity	18 (30.00%)	22 (36.67%)	0.426
Chronic kidney disease	8 (13.33%)	12 (20.00%)	0.324
Hypertension	20 (33.33%)	28 (46.67%)	0.128

SD—standard deviation.

**Table 2:** Infection characteristics among patients with OI.

Variables	Group A (*n* = 60)	Group B (*n* = 60)	*p*-Value
Type of therapy, *n* (%)			<0.001
Medical	47 (78.33%)	25 (41.67%)	
Medical + interventional	13 (21.67%)	35 (58.33%)	
Type of infection, *n* (%)			<0.001
Abscess	42 (70.00%)	18 (30.00%)	
Cellulitis	8 (13.33%)	12 (20.00%)	
Both	10 (16.67%)	30 (50.00%)	
Infection site, *n* (%)			
Perimaxillary	15 (25.00%)	12 (20.00%)	0.512
Perimandibular	20 (33.33%)	28 (46.67%)	0.128
Superficial spaces	20 (33.33%)	14 (23.33%)	0.221
Deep spaces	5 (8.33%)	6 (10.00%)	0.753
Outcomes			
Sepsis, *n* (%)	5 (8.33%)	15 (25.00%)	0.012
ICU admission, *n* (%)	1 (1.67%)	6 (10.00%)	0.049
Mortality, *n* (%)	0 (0.00%)	3 (5.00%)	0.079
Duration of hospitalization (days), mean ± SD	4.56 ± 2.12	11.84 ± 5.98	<0.001

SD—standard deviation; ICU—intensive care unit.

**Table 3:** Symptom Severity (SS) score components among patients with OI.

Variables	Group A (*n* = 60)	Group B (*n* = 60)	*p*-Value
SIRS score, *n* (%)			<0.001
0	18 (30.00%)	6 (10.00%)	
1	20 (33.33%)	8 (13.33%)	
2	12 (20.00%)	10 (16.67%)	
3	8 (13.33%)	18 (30.00%)	
4	2 (3.33%)	18 (30.00%)	
Trismus score, *n* (%)			<0.001
Normal	34 (56.67%)	12 (20.00%)	
Moderate	20 (33.33%)	18 (30.00%)	
Severe	6 (10.00%)	30 (50.00%)	
Dysphagia score, *n* (%)			0.018
Normal	12 (20.00%)	6 (10.00%)	
Mild	28 (46.67%)	18 (30.00%)	
Moderate	18 (30.00%)	30 (50.00%)	
Severe	2 (3.33%)	6 (10.00%)	
Fascial space score, *n* (%)			<0.001
Low risk	40 (66.67%)	14 (23.33%)	
Moderate risk	18 (30.00%)	28 (46.67%)	
High risk	2 (3.33%)	18 (30.00%)	
Dehydration/comorbidities, *n* (%)			0.002
None	32 (53.33%)	14 (23.33%)	
Dehydration or comorbidities	24 (40.00%)	26 (43.33%)	
Dehydration and comorbidities	4 (6.67%)	20 (33.33%)	

SIRS—Systemic Inflammatory Response Syndrome.

**Table 4:** Laboratory parameters and biomarker scores among patients with OI.

Variables	Group A (*n* = 60)	Group B (*n* = 60)	*p*-Value
WBC (×10^9^/L), mean ± SD	9.85 ± 2.14	13.72 ± 3.18	<0.001
Neutrophils (×10^9^/L), mean ± SD	6.58 ± 1.86	10.24 ± 2.64	<0.001
Lymphocytes (×10^9^/L), mean ± SD	2.12 ± 0.68	1.74 ± 0.56	0.002
dNLR, median (IQR)	2.85 (2.10–3.50)	6.20 (5.10–7.80)	<0.001
qSOFA score, median (IQR)	0.85 (0–1)	2.00 (2–3)	<0.001
MEWS, mean ± SD	3.22 ± 1.14	7.84 ± 2.36	<0.001
PIRO score, mean ± SD	6.18 ± 2.24	14.56 ± 3.78	<0.001

qSOFA—Quick Sequential Organ Failure Assessment; dNLR—derived Neutrophil-to-Lymphocyte Ratio; MEWS—Modified Early Warning Score; PIRO—Predisposition, Infection, Response, and Organ Dysfunction score.

**Table 5:** Logistic regression analysis for predicting severe OI.

Variables	Odds Ratio (95% CI)	*p*-Value
qSOFA score	5.68 (3.28–8.72)	<0.001
dNLR	4.22 (2.86–6.54)	<0.001
MEWS	6.45 (3.78–9.12)	<0.001
PIRO Score	8.45 (4.12–12.78)	<0.001

qSOFA—Quick Sequential Organ Failure Assessment; dNLR—derived Neutrophil-to-Lymphocyte Ratio; MEWS—Modified Early Warning Score; PIRO—Predisposition, Infection, Response, and Organ Dysfunction Score.

**Table 6:** ROC curve analysis of scoring systems.

Scoring System	AUC	Sensitivity (%)	Specificity (%)	*p*-Value	PPV (%)	NPV (%)
qSOFA	0.845	78.33	81.67	<0.001	52.7	93.5
dNLR	0.812	76.67	78.33	<0.001	48	92.8
MEWS	0.878	83.33	85.61	<0.001	60.2	95.2
PIRO	0.912	88.33	82.05	<0.001	56.2	96.4

qSOFA—Quick Sequential Organ Failure Assessment; dNLR—derived Neutrophil-to-Lymphocyte Ratio; MEWS—Modified Early Warning Score; PIRO—Predisposition, Infection, Response, and Organ Dysfunction score.

## Data Availability

The data presented in this study are available upon request from the corresponding author.
